# The relationship between anxiety, depression and illness perception in tberculosis patients in Pakistan

**DOI:** 10.1186/1745-0179-4-4

**Published:** 2008-02-26

**Authors:** Mohammed O Husain, Sam P Dearman, Imran B Chaudhry, Nadeem Rizvi, Waquas Waheed

**Affiliations:** 1 St. George's University of London, London, UK; 2 Lancashire Care NHS Trust, Preston, UK; 3 Pakistan Institute of Learning and Living, Research and Development, Karachi, Pakistan; 4 Jinnah Post Graduate Medical Centre, Medicine, Karachi, Pakistan

## Abstract

**Objective:**

As the rates of TB world over have increased during the past 10 years, there has been a growing awareness of depression and its role in the outcome of chronic disorders. Though depression is common in patients with TB no study as yet has examined the prevalence of depression in this group in Pakistan. We aimed to determine the presence of depression, anxiety and illness perceptions in patients suffering from Tuberculosis (TB) in Pakistan.

**Methods:**

108 consecutive outpatients with tuberculosis completed the Hospital Anxiety and Depression scale (HADS) and the Illness Perception Questionnaire (IPQ).

**Results:**

Out of 108 patients, 50 (46.3%) were depressed and 51 (47.2%) had anxiety. Raised depression and anxiety scores were associated with an increase in the number of symptoms reported (HADS Depression: r = 0.346, p = < 0.001), more serious perceived consequences (HADS Depression: r = 0.279, p = 0.004, HADS Anxiety: r = 0.234, p = 0.017) and less control over their illness (HADS Depression: r = 0.239, p = 0.014, HADS Anxiety: r = 0.271, p = 0.005).

**Conclusion:**

We found that about a half of patients in our sample met the criteria for probable depression and anxiety based on HADS score. Negative illness perceptions were clearly related to reports of mood symptoms. As depression and lack of perceived control over illness in those suffering from tuberculosis are reported to be independent predictors of poor adherence further studies to investigate their relationship with medication adherence are required.

## Introduction

Tuberculosis is a chronic infectious disease caused by Mycobacterium tuberculosis and is one of the leading causes of mortality worldwide [1,2]. Almost a third of the world's population, approximately 2 billion people are infected with M. tuberculosis [3]. There were 8.8 million new cases of TB in 2005, the highest rates per capita being in Africa (28% of all TB cases) and half of all new cases in 6 Asian countries namely Bangladesh, China, India, Indonesia, Pakistan and the Philippines [4]. Estimates suggest that 5.7 million of Pakistan's current population of 144 million suffer from TB, with 260, 000 new cases occurring each year [3].

One of the main causes of treatment failure and rise in the prevalence of TB is due to poor treatment adherence [5] The factors determining compliance with TB treatment regimes are not well understood as yet, however over the years one of the main efforts in reducing TB prevalence has been directed towards Direct Observed Therapy (D.O.T.) to enhance compliance to TB medication, disappointingly, the evidence suggests that D.O.T. shows little advantage over self-treatment [6].

The self-regulation model (SRM) [7] suggests that the illness beliefs of an individual will guide their coping strategies. The SRM hypothesizes that beliefs about the identity of the illness, the perceived consequences of the illness, the likely causes of the illness, a likely time line of how long the illness will last and the potential for control or cure [8] are the beliefs guiding the responses of illness.

Awareness about depression and its role in the outcome of chronic disorders like rheumatoid arthritis and COPD has increased over the years [9,10]. Diabetes like TB is a chronic illness and research into diabetes has indicated that psychological factors, particularly depression and the patients' perceptions about their illness predict poor glycemic control [11]. The efficacy of enhanced psychological treatments on improved diabetes self-care has been demonstrated [12].

In a United Kingdom based sample of TB patients higher rates of depression and anxiety were observed in the poorly compliant TB patients, as were more negative health beliefs. Depression and lack of perceived control were independently associated with poor adherence. Thus treating psychological problems in patients with tuberculosis may substantially improve treatment adherence, although further research is needed [13].

Karachi is the major economic hub of Pakistan and a cosmopolitan city characterized by having a very privileged to low-income population, with the latter having very little access to the health care system. The low-income population also suffers from overcrowding and malnutrition, and therefore is predisposed to developing TB [14]. Though depression is common in patients with TB, affecting up to 52% patients [15-17], no study has as yet examined the prevalence of depression and illness beliefs in Pakistan.

In this study, we aim to examine the rates of depression and anxiety, and the nature of individual illness beliefs in patients suffering from tuberculosis attending out patient clinic in Karachi, Pakistan.

## Methods

Jinnah Post graduate Medical Centre (JPMC) with 1185 inpatient beds is one of the largest teaching hospitals in Pakistan attached to Sindh Medical College, Karachi. It provides treatment services to around 880, 000 and 170 000 patients per year at the outpatient clinics and emergency departments respectively. Although based in an urban metropolis, JPMC receives patients from surrounding rural areas as well.

One day per week over a 3 month period successive Mycobacterium tuberculosis culture positive patients attending the TB clinic at the Chest disease department of JPMC were approached. In Pakistan, TB is diagnosed and treatment initiated in TB clinics in General hospitals and is often the first point of contact. Thus our sample is highly reflective of the general TB population, which will make the results generalisable to community based TB patients under treatment. Subjects were excluded if they were less than 18 years old, or if they suffered from any major psychiatric disorder other than anxiety or depression. Those with other coexisting physical illness were also excluded in order to avoid the confounding influence of these factors on psychological well being.

Whilst waiting for their consultation in the TB clinic all consecutive patients were approached and details of the study provided to them. Those giving written consent were invited to complete a questionnaire to record demographic characteristics and details of their TB (site of infection, duration of treatment). Due to low levels of literacy amongst the patients and in order to maintain uniformity questionnaires were read to all patients by gender matched trained mental health interviewers who had past experience of working with similar subjects.

The Urdu version of 14-item, self-rated Hospital Anxiety and Depression Scale (HADS) was used to record symptoms of anxiety and depression [18]. This questionnaire was designed for use in patients with physical illnesses and avoids recording details of the biological symptoms of depression that might arise as a result of the physical complaints. Subscale scores provide a measure of both anxiety and depression as continuous variables. Scores of 11 or above on the anxiety or depression subscale are taken as indicative of probable 'case ness' for either disorder.

The Illness Perception Questionnaire (IPQ) was used to record patients' personal beliefs about TB [19]. The IPQ assesses 5 dimensions of the personal beliefs relating to their illness: (1) symptom load ("Identity" subscale), (2) expected illness duration ("Timeline" subscale), (3) anticipated impact of illness – ("Consequences" subscale), (4) perceived potential for illness to be controlled/cured "Control/Cure" subscale) and (5) likely cause of the illness ("Cause" subscale). The latter cause subscale provides qualitative data and is excluded from our analysis. Culturally validated translation of IPQ into Urdu was developed based on published guidelines [20].

### Statistical analysis

Statistical analysis was performed using SPSS 13.0 for windows. After confirming that key variables were normally distributed, the degree of linear association between variables was determined using Pearson's Correlation Coefficient (r). The independent effect of the variables considered on the presence of 'caseness' disorder was assessed by binary logistic regression analysis.

## Results

### Patient Characteristics

Of the 131 patients that met inclusion criteria, 117 consented to take part in this study.

We have complete data on 108 patients of these patients. Non-participants did not differ significantly from participants in terms of demographics or illness characteristics.

The sample consisted of 86 (79.6%) females and the mean age of the total sample was 37.3 years. Majority of the sample were 86 (79.6%), were unemployed. At the time of study 63 (58.3%) subjects were married, 38 (35.2%) single, 6 (5.6%) widowed and 1 (0.9%) divorced.

Fifty of the patients (46.3%) met study criteria for depression (HAD depression score ≥ 11) and 51 (47.2%) had anxiety (HAD anxiety score ≥ 11).

### Univariate associations

HADS anxiety and HADS depression scores were not correlated (Pearson's Correlation Coefficient (r) = 0.91, p = 0.351). Raised depression and anxiety scores were associated with an increase in the number of symptoms reported, more serious perceived consequences and less control over the illness (table 1).

**Table 1:** Pearson's Correlations Coefficients (r), and p-values, showing the association between mood and illness perceptions

	*HADS Anxiety*	*HADS Depression*
***IPQ Identity***	**(r) = 0.312, p = < 0.001**	**(r) = 0.354, p = < 0.001**
***IPQ Time-line***	(r) = 0.177, p = 0.067	(r) = 0.078, p = 0.422
***IPQ Consequences***	**(r) = 0.281, p = 0.003**	**(r) = 0.306, p = 0.001**
***IPQ Control/Cure***	**(r) = 0.313, p = 0.001**	**(r) = 0.269, p = 0.005**

These associations continued to be significant when controlling for patients age, gender, marital and employment status (table 2).

**Table 2:** Partial Correlation Coefficients (r), and p-values, showing the association between mood and illness perceptions controlling for age, gender, duration of illness and pulmonary and non-pulmonary infection.

	*HADS Anxiety*	*HADS Depression*
***IPQ Identity***	(r) = 0.307, p = 0.002	**(r) = 0.346, p = < 0.001**
***IPQ Time-line***	(r) = 0.180, p = 0.068	(r) = 0.081, p = 0.413
***IPQ Consequences***	**(r) = 0.234, p = 0.017**	**(r) = 0.279, p = 0.004**
***IPQ Control/Cure***	**(r) = 0.271, p = 0.005**	**(r) = 0.239, p = 0.014**

### Regression analysis

Regression analysis was performed to identify variables associated with whether the patient was categorised as depressed or anxious (tables 3 and 4 respectively). The perception of greater symptom load was independently predictive of both depression and anxiety.

**Table 3:** A summary of the results of regression analysing factors independently associated with depression in TB patients.

*Variable*	*OR (95% C.I.)*	*Significance (p-value)*
**Gender**	1.08 (0.21–5.56)	0.925
**Age**	1.01 (0.98–1.04)	0.405
**Marital status**	0.95 (0.51–1.58)	0.848
**Employment status**	1.28 (0.25–6.57)	0.778
**IPQ Identity**	**1.21 (1.06–1.38)**	**0.005**
**IPQ Time line**	1.10 (0.90–1.34)	0.352
**IPQ Consequences**	1.12 (0.98–1.28)	0.110
**IPQ Control**	1.07 (0.95–1.20)	0.263

**Table 4:** A summary of the results of regression analysing factors independently associated with anxiety in TB patients.

*Variable*	*OR (95% C.I.)*	*Significance (p-value)*
**Gender**	0.83 (0.18–3.95)	0.816
**Age**	1.01 (0.98–1.04)	0.349
**Marital status**	1.45 (0.70–1.89)	0.594
**Employment status**	0.97 (0.20–4.63)	0.970
**IPQ Identity**	**1.16 (1.02–1.31)**	**0.021**
**IPQ Time line**	1.03 (0.94–1.12)	0.595
**IPQ Consequences**	1.09 (0.95–1.24)	0.222
**IPQ Control**	1.08 (0.96–1.21)	0.215

## Discussion

Poor treatment adherence in medical conditions has resulted in worse clinical outcomes, but also subsequent hospitalisations and increased health care costs [21].

Lately there has been a growing interest in psychiatric co-morbidity in physically ill patients and understanding of its consequences particularly poor adherence. However, to date there has been paucity of medical literature that has focused on co morbid depression and illness perception in tuberculosis.

Current study aimed to look at the prevalence of depression, anxiety and negative illness beliefs in patients suffering from tuberculosis. Prevalence of depression (46%) and anxiety (47%) in these TB patients is further higher than already reported mean prevalence of anxiety and depression in Pakistan found to be around 34% (range 29–66% for women and 10–33% for men) in community based population [22]. However, the results demonstrate that raised depression and anxiety scores were associated with an increase in the number of TB symptoms reported, more serious perceived consequences and less control over the illness. This is important because these factors may contribute to poor compliance with TB medication.

One of the limitations of the study is that it is cross sectional in design with no control group thus casual relationships can only be inferred. However one of the strengths of this study is that we have used validated and recognised measures. Though, HADS a self-report measure has been previously validated in Pakistan a limitation of the study may be that we did not use a gold standard psychiatric interview. The sample size is small but out of the eligible patients we were able to recruit a considerably high proportion, which means that the sample was representative and results generalisable.

These findings highlight the benefits of regular screening for depression in the medical outpatient clinic particularly TB clinics. Treating depression and working with the patients to improve their illness perceptions may help improve treatment adherence, disease outcomes and improve overall patient management. Taking into consideration low literacy levels and other cultural barriers new treatment strategies should incorporate psycho education and cognitive behavioural therapy to achieve these treatment outcomes. Lately there have been randomised control trials undertaken for psychological treatments of depression in developing countries with encouraging results. The content and structure of these interventions can be modified to address not only depression but also issues around adherence to treatment and illness perception as well [23].
